# Phased Array Antenna Analysis Workflow Applied to Gateways for LEO Satellite Communications

**DOI:** 10.3390/s22239406

**Published:** 2022-12-02

**Authors:** Irene Merino-Fernandez, Sunil L. Khemchandani, Javier del Pino, Jose Saiz-Perez

**Affiliations:** Institute for Applied Microelectronics, University of Las Palmas de Gran Canaria (ULPGC), Parque Cientifico Tecnologico, 35017 Las Palmas de Gran Canaria, Spain

**Keywords:** gateway, Low Earth Orbit constellations, patch antenna, phased array, satellite communications

## Abstract

Nowadays, mega-constellations of Low Earth Orbit (LEO) satellites have become increasingly important to provide high-performance Internet access with global coverage. This paper provides an updated comparison of four of the largest LEO mega-constellations: Telesat, SpaceX, OneWeb and Amazon. It describes the gateway design workflow from the patch antenna to phased array analysis. Patch antennas are developed for both transmission and reception after a thorough examination of the four systems. The results of electromagnetic simulation using Advanced Design Software (ADS) Momentum are shown, including their radiation pattern. Finally, a model of the gateway phased array using SystemVue is obtained using hexagonal, circular, and square arrays. According to the required effective isotropic radiated power (EIRP) and gain, the antenna sizes for the four constellations are estimated. As an example, for SpaceX constellation, a reception antenna with 8910 radiating elements using a hexagonal distribution with a gain of 46.9 dB and a sensitivity of −113.1 dBm was obtained.

## 1. Introduction

Low Earth Orbit (LEO) is presented as a complement to 5G communications, improving the bandwidth of mobile communications, giving global coverage, and allowing a wide range of Internet of Things (IoT) applications or high trust communications. 5G plays a key role in the development of high-density sensor networks. The 5G system increase data rates by 10 times that of the traditional Long-Term Evolution networks, to an average of 10 Gbps with a 1 ms round-trip latency. This high bandwidth can accommodate many connected devices per unit area under the IoT framework [1].

High data rate services are not available in rural places or onboard ships and aircrafts since terrestrial networks do not cover the whole world. SATCOM and SATCOM-on-the move (SOTM) are essential to achieve communications with global coverage. The idea of delivering internet connectivity from space has made a significant resurgence. Although some proposals for LEO mega-constellations of satellites emerged in the 90s, it is not until now that these projects are being implemented. Technical innovations of the past decades, such as increased performance resulting from the use of digital communication payloads, advanced modulation schemes, multi-beam antennas, and sophisticated frequency reuse schemes, as well as overall cost reductions from advanced manufacturing processes and reduced launch costs, have enabled the launch of mega-constellations of low-cost satellites [2].

Mega-constellations refer to a constellation composed of hundreds or thousands of satellites orbiting the Earth [3]. In particular, LEO refers to orbits between 500 and 1000 km, having reduced path loss and delay compared to Geostationary Earth Orbit (GEO), which is located 35,786 km above Earth’s equator [4]. LEO is presented as a complement to 5G communications, improving the bandwidth of mobile communications, giving global coverage and allowing a wide range of Internet of Things (IoT) applications or high trust communications. This system could also give internet service to remote areas [5].

**Figure 1:**
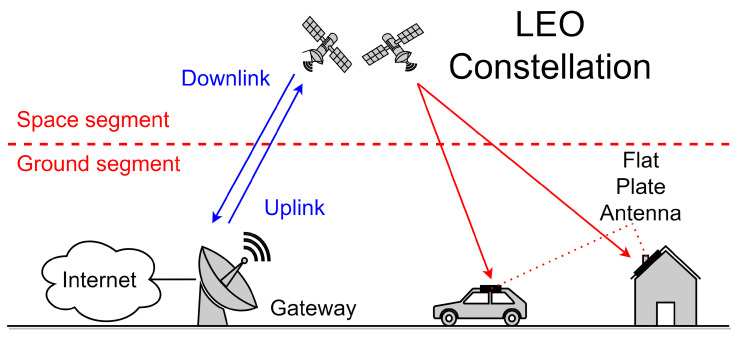
Satellite communications system.

Figure 1 illustrates a typical satellite communication system. The space segment consists of the satellites, and the ground segment will be composed of three different types of elements: the Tracking, Telemetry and Commands (TT&C) stations, the gateways, and the user terminals. The TT&C stations are responsible for monitoring the satellite in orbit and will be scarce in number and distributed across the world. The gateways are used to give users’ terminals access to the Internet through the satellites. Contrary to what happens with conventional satellite systems, in this type of system a large number of gateway antennas distributed across the world near or co-located with Internet peering points are required.

The development of large LEO and Medium Earth Orbit (MEO) constellations, planned by companies such as SpaceX, Telesat, OneWeb and Amazon will require ground terminals able to track multiple satellites working at Ku-Ka-bands. Parabolic dish antennas have been the defacto choice for SATCOM Earth antennas. They provide benefits such as high performance, low power consumption, and low cost. However, they have limitations, such as weight, size, lower efficiency [6] and its mechanically pointing. To communicate with LEO satellites, where orbital movement is faster than GEO, the pointing needs to change quickly.

This is where phased array antennas have emerged as a solution. Relative phases of signal feeding of the patch antenna are varied to alter the radiation pattern so that the phased array points in a specific direction electronically without the physical movement of the antenna. Moreover, dish antennas become inefficient and costly when tens or hundreds of beams are required, as is the case of current LEO constellations where many tens of beams per satellite for Mobile Satellite Services (MSS) and hundreds of beams per satellite for wideband data systems are required [7]. Because of their LEO orbit, these antennas must scan over very wide angles simultaneously with multiple beams, thus making the phased arrays the only competitive solution [8].

Array antennas are developed as a key architecture for wireless communication systems, with Multiple Input Multiple Output (MIMO) antenna arrays included in the standards for cellular and wireless local area networks. In next-generation High Throughput Satellite (HTS) communications, these active antenna arrays will play a fundamental role [6]. Other advantages of this technology are the increase in total power due to the combined effect of the signal from the individual antennas and the improvement in reception sensitivity due to the higher signal-to-noise ratio resulting from increasing the number of individual antennas.

This paper describes a novel workflow based in PathWave System Design (SystemVue) for modelling and design phased array antennas for LEO satellite communications gateways. The objective of this work is to study gateway ground station phased arrays for four constellations of satellites: SpaceX, Telesat, OneWeb and Amazon. After a review of the technical characteristics of these systems, two rectangular patch antennas are designed, one for transmission and one for reception, and then the number and spatial configuration of the radiating elements required to provide the service are defined. This paper is structured as follows: Section 2 reviews the basic theory to understand phased array antennas. Section 3 gives an overview of the four systems: SpaceX, Telesat, OneWeb and Amazon. Section 4 shows how patch antennas are designed and the results of electromagnetic simulation for the gateway using Keysight PathWave Advanced Design Software (ADS) Momentum. Section 5 discusses the modelling of the phased array using SystemVue. Finally, Section 6 presents the conclusions, summarising the results.

## 2. Array Theory

**Figure 2:**
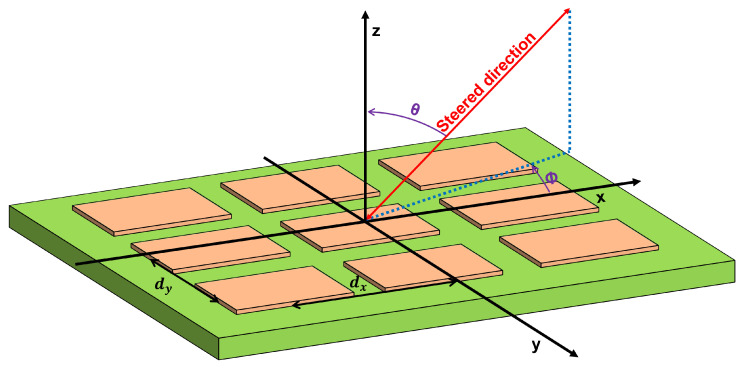
One-dimensional phased array diagram.

A phased array antenna is a computer-controlled antenna array that produces a beam of radio waves that can be electrically directed in various directions without moving the antennas [9]. The working principle is based on phase shifting and coherent combining signals from different antennas in a process called beamforming, as shown in Figure 2.

In the case of a ground station antenna, a two-dimensional phased array is chosen. These alter pointing angles in terms of elevation θ, or scanning angle, and azimuth ϕ, or beam direction. Phase differences are defined by (1) and (2), where ΔΦx is the phase difference between the array *x*-axis elements, ΔΦy is the phase difference between *y*-axis elements, λ is the wavelength, $d_{x}$ is the distance between *x*-axis elements and $d_{y}$ is the distance between y-axis elements [10].

(1) $$\Delta \Phi x=\frac{2\pi }{\lambda }d_{x}sen\theta cos\phi$$

(2) $$\Delta \Phi y=\frac{2\pi }{\lambda }d_{y}sen\theta sen\phi$$

For this type of application, antenna arrays are generally composed of patch antennas due to their low profile, low cost, light weight and ease of connection to solid state devices. They consist of a thin metallic layer bonded to a grounded dielectric substrate and can have any geometric shape such as rectangles or circles [11].

**Figure 3:**
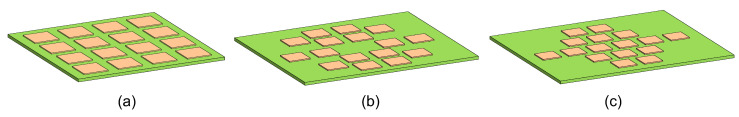
(**a**) Square. (**b**) Circular. (**c**) Hexagonal distribution for phased array antenna design.

Phased array antennas consist of a large number of radiating elements, in some cases on the order of thousands of elements, and can be distributed in different geometrical shapes, such as square, circular or hexagonal (see Figure 3). The geometrical distribution of the patch antennas will determine the radiation pattern of the whole array.

**Figure 4:**
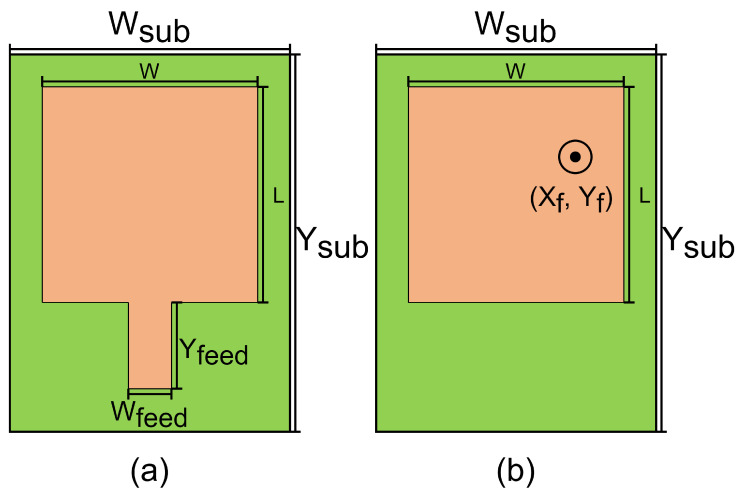
Methods to feed patch antennas: (**a**) using microstrip, (**b**) using feeding point.

To design the antenna, some key parameters should be tackled. First is the radiation pattern, which is the evaluation of the far-field amplitude for all possible angular positions of the antenna, and is normalized to the maximum value [9]. Second is the matching bandwidth of the antenna when connected to the transmitter or receiver, which is measured by the input impedance or reflection coefficient [9]. This parameter depends on how the antenna is fed. As shown in Figure 4, there are two methods to feed the patch antenna:

1. Through a microstrip line (see Figure 4a): This method is used when the circuit that feeds the patch antenna is in the same layer as the antenna. The dimensions of the microstrip line that feeds the antenna are $W_{f}$ and $Y_{f}$.
2. Through a point inside the patch antenna itself (see Figure 4b): This method is used when the circuit that feeds the patch antenna is in a different layer as the antenna. This point corresponds to ($X_{f}$, $Y_{f}$).

The last parameter that is analysed is the gain, which is the maximum of the gain function. The gain function G(θ,φ) is the ratio between the power radiated by the antenna into a direction P(θ,φ) and the total accepted power in the same direction by an isotropic radiator Pin/4π [9], as observed in (3). This function does not consider the impedance mismatch on the antenna terminals [9].

(3) $$G\left(\theta ,\varphi \right)=\frac{P(\theta ,\varphi )}{Pin/4\pi }$$

In the case of the gateway phased array with thousands of patch antennas, due to size restrictions and the high amount of associated circuitry which is placed in a different layer than the patch antenna, the feed of the patch antenna is usually made by a point inside the patch antenna itself. The use of circular polarization would complicate its implementation, adding an extra λ/4 phase shift. This is the reason why this study has been carried out with linear polarization and feeding through a point inside the patch antenna itself.

## 3. System Architecture

Today many mega-constellations are already operating, such as Starlink from SpaceX [12], Lightspeed from Telesat [13] or WorldVu from OneWeb [14]. Recently, Amazon has received Federal Communications Commission (FCC) approval for its satellite mega-constellation project, Kuiper [15]. Moreover, the Spanish and China governments have started projects: Startical, for air traffic control by the hand of Enaire [16] and GW from China Satellite Network Group [17].

There are some studies about LEO satellite constellations from SpaceX, Telesat, OneWeb and Amazon. In [2,18] a comparison of the four systems is made from a technical point of view, estimating the throughput and the number of gateways. In [19] a comparison of OneWeb and SpaceX systems is conducted, verifying the impact of beam coverage on the performance of LEO satellite systems.

This section makes a brief study of the four gateway systems for the constellations SpaceX, Telesat, OneWeb and Amazon. They are described according to their FCC filings as of September 2022, including pending changes.

### 3.1. SpaceX’s System

The Starlink system of the SpaceX company is close to being operational because it has several hundreds of its satellites in orbit. Moreover, the beta test has started too in many countries. This constellation will have 4408 satellites, according to their files of FCC [20,21,22,23], and had its first launch in 2019.

This system has eight technically identical 1.5 m parabolic antennas in each gateway. These antennas will communicate with satellites visible on the horizon over a minimum elevation angle, which is 25 degrees [24]. For gateway communications, this constellation will use K-band frequencies for: the uplink (27.5–30 GHz), and the downlink (17.8–19.3 GHz) [2].

### 3.2. Telesat’s System

Lightspeed is the mega-constellation of the Canadian satellite communications company Telesat. According to FCC filings, it will be developed in two phases: an initial stage, with 298 satellites, and a second phase, having 1671 satellites [25,26]. It is scheduled to begin services in 2023 [27], having launched the first satellite in 2018 [28].

Satellites will connect to earth stations with four steerable spot beams with a minimum elevation of 10 degrees [29]. Their gateways will use K-band in the downlink, from 17.8 GHz to 20.2 GHz, and from 27.5 GHz to 30 GHz in the uplink [2].

### 3.3. OneWeb’s System

According to FCC filings, the global communications company OneWeb has planned to launch the WorldVu constellation of satellites in two phases [30,31,32], with its first launch in 2018. The initial phase is composed of 716 satellites, and the second phase is focused on covering Earth regions with a higher population, adding 5656 satellites to the constellation [18]. The services will start in 2023, due to a pause caused by economic problems [14].

Antennas between 2.4 and 3.5 m will be used in gateway earth stations. Each satellite will have assigned at least two gateways, and a supplementary antenna for handovers. They expect to have a minimum of five gateway earth stations in the USA, building the initial network using existing stations. These stations are also responsible for the transmission and reception of satellite payload control channels and gateway link power control. Communication with satellites is only allowed after reaching the elevation angle of 15 degrees [31]. The gateway of OneWeb will use the K-band in both uplink (27.5–30 GHz) and downlink (17.8–19.3 GHz) [2].

### 3.4. Amazon’s System

In 2019, Amazon announced their LEO mega constellation of satellites Kuiper, having their filing in an approved state [33]. The set-up of this constellation will be made in 5 phases, launching 3236 satellites. They have not yet launched any satellites, planning their first launch for the end of 2022.

The elevation of the antennas, which sizes from 1 m to 2.4 m, will be no less than 20 degrees, being unable to access Kuiper’s network below these angles. Each satellite will have access to two different gateway earth stations, reducing interferences and achieving the throughput of the system. These gateways will use the K-band in both uplink (27.5–30 GHz) and downlink (17.7–20.2 GHz) [33].

### 3.5. Comparative Assessment

Once the constellations’ systems have been described, a comparison is made. The frequency assignment is shown in Table 1 and Table 2 [24,29,31,33], where ${\#}_{CH}$ is the number of channels and † is for shared frequencies. In the Telesat system, the lower and upper Ka-band spectrum is shared between the user and the gateway links, the number of beams and the bandwidth per beam being reconfigurable [2].

Regarding the parabolic antennas, the most important values are summarized in Table 3. It should be noted that more than one antenna is needed to make an earth station, so they will be occupying a large surface, considering the diameter and the above ground level [24,29,31,33]. The values of the effective isotropic radiated power (EIRP), maximum gain and path distance are taken from [2,33,34]. The minimum value of elevation needed to communicate with the satellites in LEO is obtained from [24,29,31,33].

## 4. Patch Antenna Design

This section explains the design procedure of the transmission and reception patch antennas. First, the substrate is selected and then its dimensions are calculated. Finally, the antenna feeding point is determined.

The most important parameters of the substrate are its dielectric constant ($\varepsilon_{r}$) and its thickness (*h*), which are determined considering the operating frequency (*f*). Later, using (4)–(7) [35] the length (*L*) and width (*W*) of the antenna can be calculated, where $L_{eff}$ is the effective length, ΔL is the length extension, and $\varepsilon_{eff}$ is the effective dielectric constant of the substrate.

(4) $$W=\frac{c}{2f}\sqrt{\frac{2}{\varepsilon_{r}+1}}$$

(5) $$L=L_{eff}-2\Delta L$$

(6) $$L_{eff}=\frac{c}{2f\sqrt{\varepsilon_{eff}}}$$

(7) $$\Delta L=0.412h\frac{\left(\varepsilon_{eff}+0.3\right)\left(\frac{W}{h}+0.264\right)}{\left(\varepsilon_{eff}-0.258\right)\left(\frac{W}{h}+0.8\right)}$$

**Figure 5:**
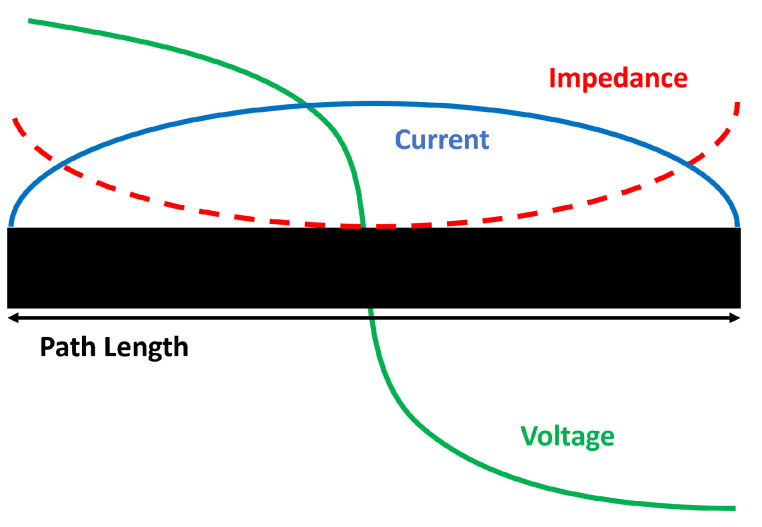
Impedance distribution in a patch antenna [36].

Next, the feeding point was calculated. In this case, the beamformer chips are placed in a different layer than the patch antenna. For a large patch antenna array, using this feeding approach we can reduce the beamforming. To choose our feeding point, the patch antenna impedance distribution must be considered. As shown in Figure 5, the impedance is at the maximum at the edges and minimum at the centre. By applying (8) and (9) the exact position (Xf and Yf) of the 50 Ω impedance point to feed the patch antenna can be calculated [35,36]. Once this point was calculated, it was adjusted by simulations.

(8) $$X_{f}=\frac{L}{\sqrt{\varepsilon_{eff}}}$$

(9) $$Y_{f}=\frac{W}{2}$$

### 4.1. Design of Transmission Patch Antenna

**Figure 6:**
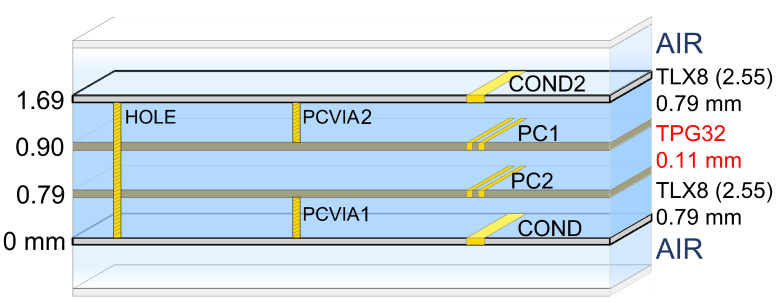
ADS model of the selected substrate for the transmission antenna composed of two TLX-8 substrates glued back-to-back with TPG-32 pre-impregnated material.

The frequency band of the transmission antenna is centred at 28.75 GHz and ranges from 27.5 GHz to 30 GHz.Figure 6 shows the ADS substrate model of the six-layer Printed Circuit Board (PCB) used for electromagnetic (EM) simulations. The top layer (COND2) is used for the patch antenna, and the bottom layer (COND) is used for the beamforming circuitry. PC1 and PC2 are the ground planes of the top and bottom layers, respectively, and the dielectric between COND2-PC1 and COND-PC2 is a Taconic TLX-8 fibreglass substrate with 2.55 relative permittivity ($\varepsilon_{r}$) and 0.79 mm height. PC1 and PC2 are glued back-to-back with a 0.1143 mm thickness TPG-32 pre-impregnated material. PCVIA1 and PCVIA2 are the vias to connect the conductor (COND2 and COND) layers to their respective ground planes (PC1 and PC2). A third via (HOLE) is needed to connect the antenna with the beamforming circuitry [37].

**Figure 7:**
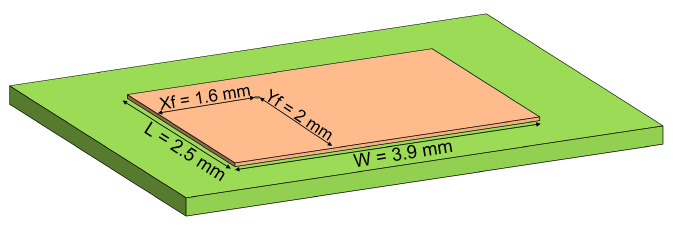
Transmission antenna layout diagram.

Using (4)–(9), the patch antenna dimensions were calculated. The results are summarized in Figure 7. The width and length of the antenna are 3.9 mm and 2.5 mm, respectively, and the feeding point for a 50 Ω input impedance is located at $X_{f}$ = 1.6 mm and $Y_{f}=$ 2 mm.

**Figure 8:**
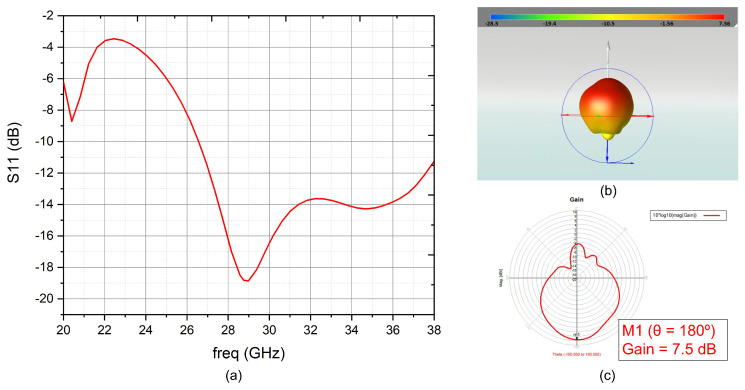
(**a**) $S_{11}$ for the transmission patch antenna (**b**) 3-D transmission antenna radiation pattern (**c**) 2-D transmission antenna radiation pattern.

The simulated reflection coefficient ($S_{11}$) is shown in Figure 8a. The reflection coefficient is better than −10dB over the whole frequency band (from 27.5 GHz to 30 GHz) and its minimum is located at the centre of the band. As shown in Figure 8b,c, the maximum gain is 7.5 dB.

### 4.2. Design of Reception Patch Antenna

**Figure 9:**
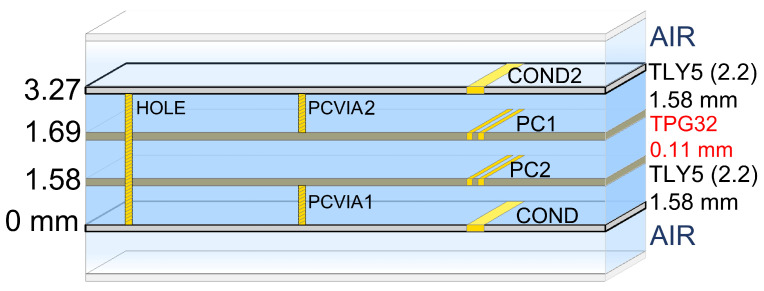
ADS model of the selected substrate for the reception antenna composed of two TLY-5 substrates glued back-to-back with TPG-32 pre-impregnated material.

The gateway reception frequency bands for LEO constellations are slightly different: SpaceX and OneWeb are from 17.8 GHz to 19.3 GHz and are centred at 18.55 GHz, Telesat is from 17.8 GHz to 20.2 GHz and is centred at 19 GHz while Amazon is from 17.7 GHz to 20.2 GHz and is centred at 18.95 GHz. Since the frequencies are not very different, the same substrate will be used for all of them. In this case, the chosen substrate was Taconic TLY-5 with $\varepsilon_{r}=$2.2 and h= 1.58 mm. To glue the two layers, the same pre-impregnated material as for the reception antenna is used (TPG-32). Figure 9 shows the ADS substrate model of the six-layer PCB used for EM simulations.

**Figure 10:**
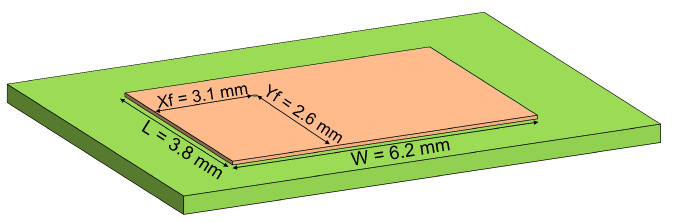
Reception antenna layout diagram.

Figure 10 shows a physical representation of the reception antenna. In this case, the width and length of the antenna are 6.2 mm and 3.8 mm, respectively, and the feeding point for a 50 Ω input impedance is located $X_{f}$ = 3.1 mm and $Y_{f}$ = 2.6 mm. As the frequencies of the four considered systems are not very different, there were no major differences between the results.

**Figure 11:**
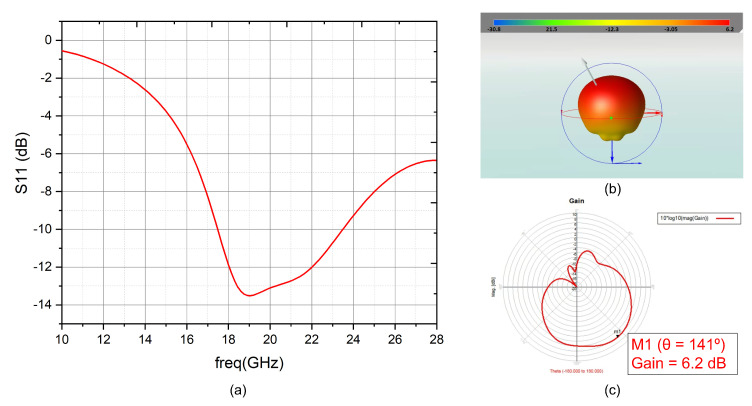
(**a**) $S_{11}$ for the reception patch antenna (**b**) 3-D reception antenna radiation pattern (**c**) 2-D reception antenna radiation pattern.

In Figure 11a is shown the reflection coefficient, which is below −10 dB over all the frequency bands selected. The antenna’s radiation pattern is illustrated in Figure 11b,c, obtaining a maximum gain of 6.2 dB.

## 5. Phased Array Modelling

**Figure 12:**
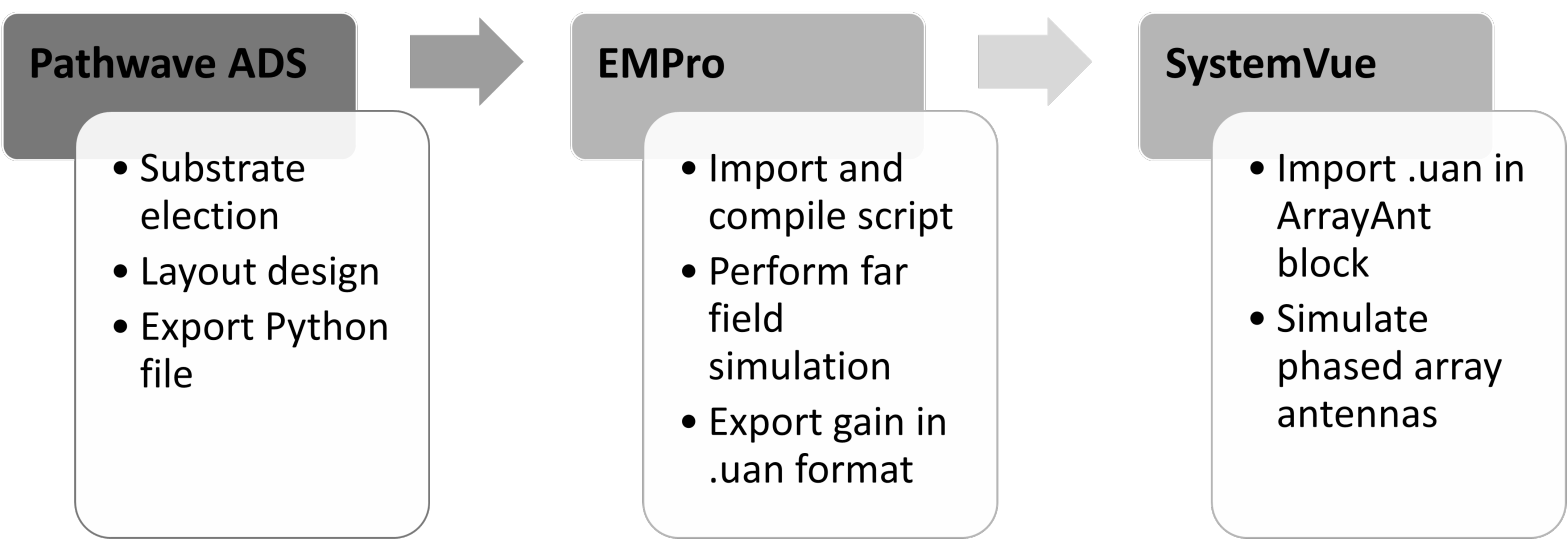
Workflow to model a large phased array using SystemVue from the patch antenna electromagnetic simulation.

This section describes how to model a large phased array using SystemVue from the patch antenna electromagnetic results using Figure 12 workflow.

Once the antennas were designed and EM simulated using PathWave ADS, a file with the radiation pattern using a python script is obtained. EMPro was used to import, compile and run a far field simulation from the file with the geometric information of the antenna. The results were exported to an User-Defined Antenna (.UAN) file containing the gain pattern, separated in a theta and phi component, with the associated phase information. This file was imported into SystemVue to simulate a large phased array. For each LEO constellation, the number of radiating elements were calculated. For transmission, this number has been calculated as a function of its EIRP, and for reception, as a function of the required gain.

### 5.1. Transmission Phased Array Modelling with SystemVue

The EIRP values specified in Table 3 are the key parameters for estimating the number of transmission gateway antenna elements. A theoretical calculation of EIRP in dBW can be conducted using (10), where *N* is the number of elements of the array, $G_{e}$ and Γ are the gain in dBi, and the reflection coefficient of each radiating element, and $P_{in}$ is the radiofrequency (RF) power input to each patch antenna, in dBm. This equation does not consider the electromagnetic interaction between the radiating elements, so we must use Systemvue to better estimate the gateway EIRP.

(10) $$EIRP=10log\left(N\right)+G_{e}{+10log(1-|\Gamma |}^{2}+Pin-30$$

**Figure 13:**
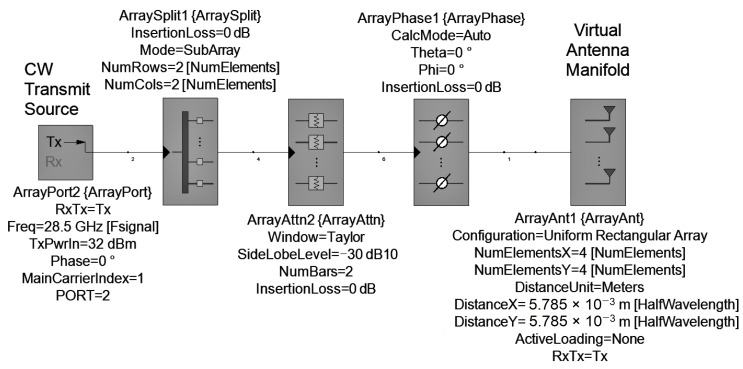
Transmission phased array model.

SystemVue schematic for transmission is shown in Figure 13. To simplify the calculations, it was assumed that the components have no insertion loss. A description of each component is summarized below:

- ArrayPort: it defines the operation frequency (Freq) and the input power of the phased array (TxPwrIn) in dBm. According to (11), TxPwrIn depends on the number of array elements (*N*), and the power output of each patch antenna ($P_{patch}$) in dBm, which we have assumed to be 20 dBm. For example, for a 2 × 2 array, is calculated a TxPwrIn of 26 dBm.
  (11) $$TxPwrIn=P_{patch}+10logN$$
- ArraySplit: it splits equally TxPwrIn power into *N* radiating elements.
- ArrayAttn: with this weighting attenuator we can achieve low peak sidelobes to concentrate all gain in the main lobe. In this case, a Taylor weighting was used [38].
- ArrayPhase: this component is used to apply the phase shifts.
- ArrayAnt: this block defines the number of radiation elements and their spatial distribution. The separation between each element (DistanceX and DistanceY) is 0.55λ. In this component, the gain pattern of each element was imported using the .UAN file obtained from EMpro.

**Figure 14:**
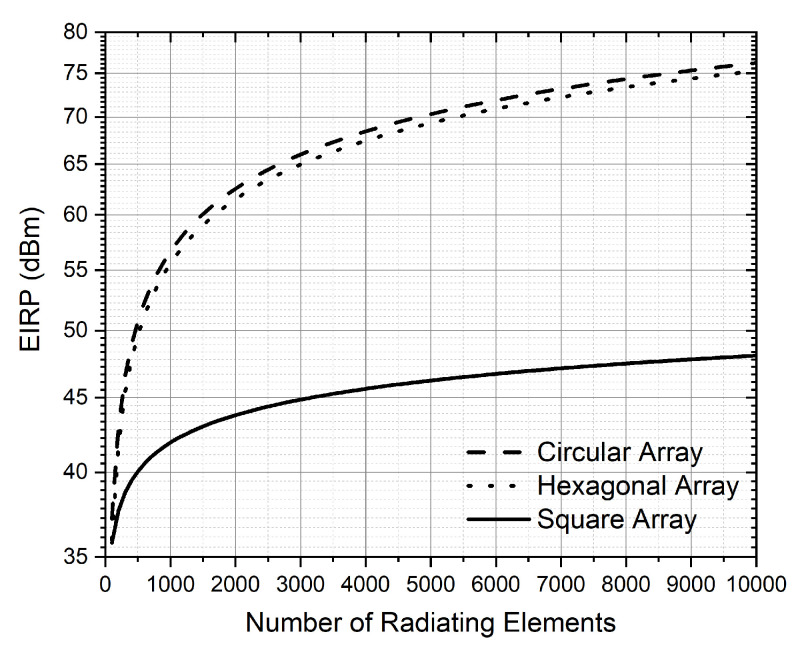
EIRP vs. number of radiating elements for transmission obtained with Systemvue.

SystemVue simulations were run by increasing the number of elements while keeping simulation time reasonable. From approximately 1K elements, the simulation time was very long. For this reason, to obtain the EIRP for a large number of radiating elements, the simulation data were extrapolated and fitted with (12)–(14) in dBW. These Equations are for circular, hexagonal and square array configurations and they are depicted in Figure 14. For a circular array, we obtain the same EIRP but with fewer radiating elements compared to the square and hexagonal configuration. In terms of saving space, the circular array is the most appropriate.

(12) $$EIRP_{circular}=8.4953ln\left(N\right)-2.0311$$

(13) $$EIRP_{hexagonal}=8.5584ln\left(N\right)-3.5192$$

(14) $$EIRP_{square}=2.6718ln\left(N\right)+23.464$$

Table 4 shows the number of radiating elements for each LEO constellation using circular configuration. An RF power input of 20 dBm is assumed for each patch antenna. Theoretical calculations (10) and those obtained through simulations (12) have been included. As expected, there is a difference between the results obtained by (10) and (12). As stated above, this is because, in theoretical calculations, the electromagnetic interaction between the radiating elements is not taken into account. Considering the logarithmic relationship between EIRP and N, Telesat needs a huge number of elements. This number can be reduced by increasing the input power of the signal applied to each patch antenna.

**Figure 15:**
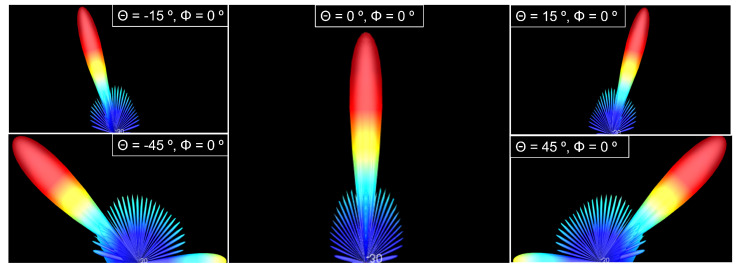
Radiation pattern of the transmission phased array for circular shape configuration and 625 elements.

A principle of the phased array is that it is electronically steerable by changes on feeding relative phases and focusing all power on the principal beam. To verify this principle, elevation angle (Θ) was swept between −45° and 45° for a circular shape-phased array with 625 elements. As observed in Figure 15, all the power is concentrated on the principal beam.

**Figure 16:**
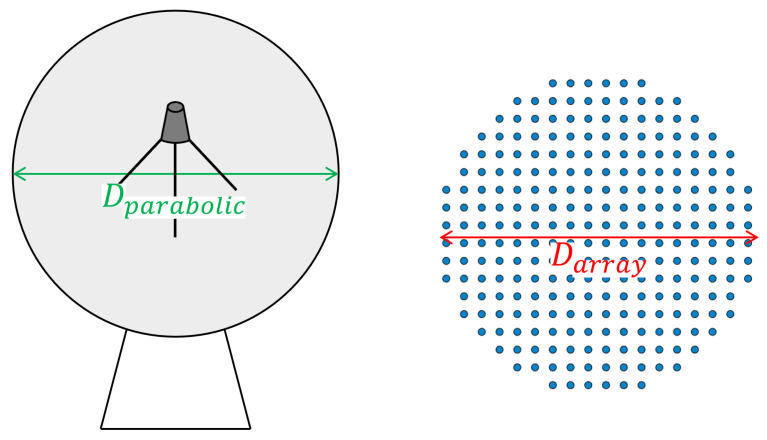
Reference parameters used for calculating the area used in transmission.

As already mentioned, one of the advantages of phased arrays over parabolic antennas is the surface area used. Table 5 and Table 6 compares the area of the two solutions, calculated using Equation (15), where *D* is the diameter of the antenna, represented in Figure 16. It should be taken into account that phased array thickness is about a few centimetres, while parabolic antennas are higher than 1 m.

(15) $$A_{circle}={\left(\frac{D}{2}\right)}^{2}\pi$$

### 5.2. Reception Phased Array Modelling with SystemVue

The receiver was simulated using SystemVue to obtain its gain versus the number of radiating elements. The gain values specified in [31,33,39,40] are the target of this section. A theoretical calculation of reception gain ($G_{array}$) in dB can be conducted using (16), where *N* is the number of radiating elements and $G_{e}$ and Γ are the gain in dBi, and the reflection coefficient of each radiating element.

(16) $$G_{array}=10log\left(N\right)+G_{e}{+10log(1-|\Gamma |}^{2})$$

**Figure 17:**
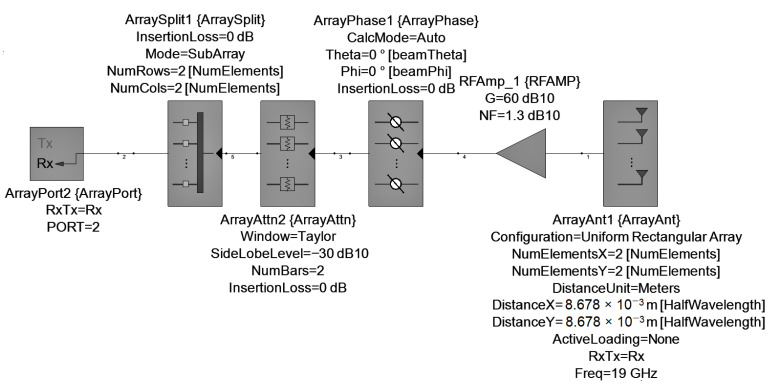
Reception phased array model.

The schematic used to model the receiver using SystemVue is shown in Figure 17. Most of the components are the same as in the case of transmission except for the RFAMP, which models the RF frontend. A gain of 60 dB and a Noise Figure (NF) of 1.3 dB is assumed for the receiver frontend. ArrayAnt component is configured to 19 GHz.

To model the receiver, the sensitivity (*S*) in dBm for each constellation is calculated using (17), where $EIRP_{sat}$ is the effective isotropic radiated power of the satellite in dBm, $L_{TM}$ is the transmission medium loss dB, $G_{e}$ is the gain of one radiating element in dBi and FSPL is the free space path loss in dB, described by (18) (in dB), where λ is the wavelength, and *R* is the path length in m. In this case, the working frequency is set to 19 GHz in Telesat and Amazon, and 18.55 GHz in SpaceX and OneWeb. The gain of each radiating element is 6.2 dB. Table 7 shows the sensitivity calculation for each constellation. The OneWeb system requires the minimum sensitivity (−114 dBm). To ensure the reliability of the system, a sensitivity of −120 dBm is assumed in the simulations.

(17) $$S=EIRP_{sat}+FSPL-L_{TM}+G_{e}$$

(18) $$FSPL=10log{\left(\frac{\lambda }{4\pi R}\right)}^{2}$$

**Figure 18:**
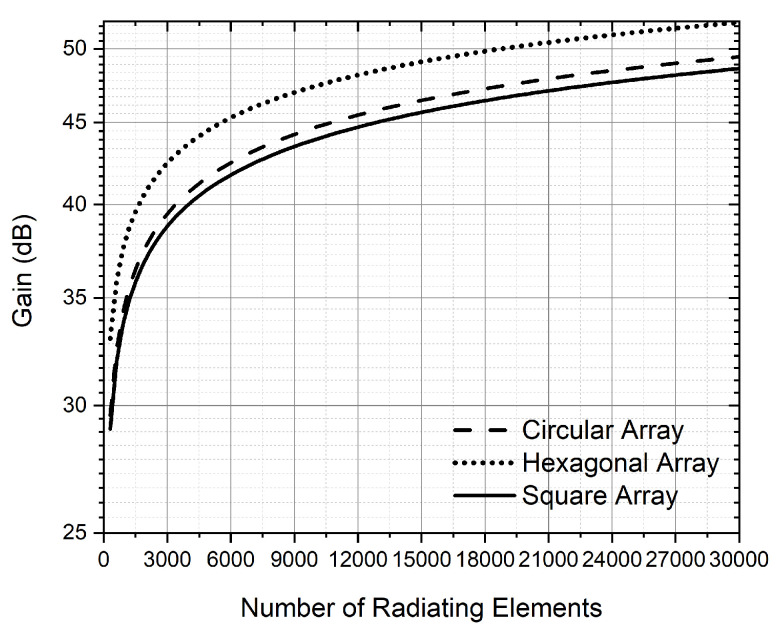
Antenna gain vs. number of radiating elements for reception obtained with Systemvue.

As in the case of transmission, due to simulation time constraints, SystemVue simulations were run approximately to 1K elements. To obtain the gain for a large number of radiating elements, the simulation data were extrapolated and fitted with (19)–(21) in dB. These equations are for circular, hexagonal, and square array configurations, and they are represented in Figure 18. For a hexagonal distribution, we obtain the lower number of radiated elements for the same gain compared to the circular and square configuration.

(19) $$Gain_{circular}=4.3188ln\left(N\right)+4.9112$$

(20) $$Gain_{hexagonal}=4.1123ln\left(N\right)+9.535$$

(21) $$Gain_{square}=4.2642ln\left(N\right)+4.646$$

Table 8 shows the number of radiating elements for each LEO constellation using hexagonal configuration modelled using (20). All reception gains are based in FCC filings of the constellations: SpaceX’s gain is based on [39], Telesat’s gain is based on [40], OneWeb’s gain is based on [31] and Amazon’s gain is based on [33]. As expected, there is a difference between the results obtained by (16) and (20). This is because in theoretical calculations, the electromagnetic interaction between the radiating elements is not taken into account. In some cases, a huge gain is required, so a large number of elements is needed.

**Figure 19:**
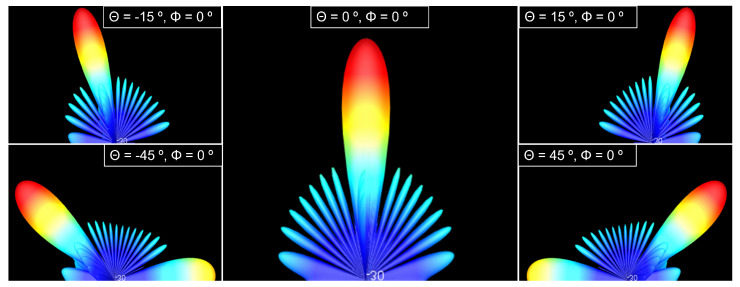
Radiation pattern of the reception antenna for hexagonal shape configuration, 625 elements and different elevations.

Figure 19 shows the radiation pattern of a 625 elements hexagonal phased array for different elevation (Θ) angles.

**Figure 20:**
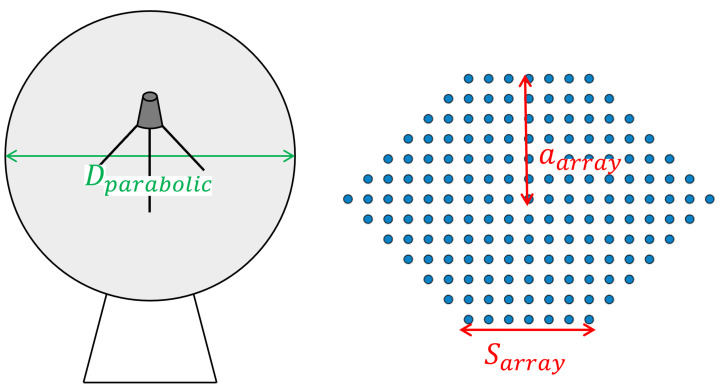
Reference parameters used for calculating the area used in reception.

Table 9 and Table 10 shows a comparison of the area used by parabolic and phased array antennas. In this case, as the hexagonal distribution was chosen, the area of the phased array $A_{hexagonal}$ was calculated using (22) and Figure 20, where *S* is the length of the side of the array and *a* is the apothem, corresponding to $S_{array}$ and $a_{array}$ in the Figure 20, respectively.

(22) $$A_{hexagonal}=\frac{6\times S\times a}{2}$$

## 6. Conclusions

LEO satellite mega-constellations are becoming increasingly important for providing high-performance Internet access with global coverage. The position of the LEO satellites is constantly changing, and phased array antennas allow orientation to be adjusted without any physical movement. They also allow multiple satellites to be used simultaneously. Consequently, the operating costs are greatly reduced compared to conventional parabolic antennas, as the space requirements are significantly reduced, and the installation and maintenance costs are also lower [8]. This paper introduces a new workflow for modelling and designing large phased array antennas for LEO satellite communication gateways. Phased array antennas from SpaceX, Telesat, OneWeb, and Amazon were developed using this methodology. Originally, patch antennas were developed for both transmission and reception. Various phased array configurations were then tested to determine the optimal physical layout and number of radiating elements. The transmission antenna was modeled by using a circular distribution, and the reception antenna with a hexagonal distribution. The specific values are summarized in Table 11.

## Figures and Tables

**Table 1:** Summary of the uplink frequency allocation.

	Gateway Uplink Links			
	${\mathrm{BW}}_{\mathrm{CH}}$	${\#}_{\mathrm{CH}}$	${\mathrm{BW}}_{\mathrm{TOT}}$	k
SpaceX	500	8	4000	1
Telesat	†	†	4200	2
OneWeb	250	16	4000	1
Amazon	50	50	2500	1
	MHz	-	MHz	-

**Table 2:** Summary of the downlink frequency allocation.

	Gateway Downlink Links			
	${\mathrm{BW}}_{\mathrm{CH}}$	${\#}_{\mathrm{CH}}$	${\mathrm{BW}}_{\mathrm{TOT}}$	k
SpaceX	250	9	2250	1
Telesat	†	†	3600	2
OneWeb	155	16	2480	1
Amazon	100	23	2300	1
	MHz	-	MHz	-

**Table 3:** Gateway antennas.

	Uplink Antenna				Downlink Antenna			
Satellite	SpaceX	Telesat	OneWeb	Amazon	SpaceX	Telesat	OneWeb	Amazon
Diameter (m)	1.5	1.5	3.5	2.4	1.5	1.5	3.5	2.4
Above ground level (m)	1.95	3.2	4.55	-	1.95	3.2	4.55	-
EIRP (dBW)	68.4	75.9	63.2	62.3	39.44	38	30.6–39	36
Max Gain (dBi)	41	31.8	-	53.1	46.9	45.5	51.5	50.2
Elevation (deg)	25	10	15	20	25	10	15	20
Path distance (km)	2439	1504	1684	590	2439	1504	1684	590

**Table 4:** Number of radiating elements for transmission using circular configuration. An RF power input of 20 dBm has been assumed for each patch antenna.

Constellation	SpaceX	Telesat	OneWeb	Amazon
Operation Frequency (GHz)	28.5	28.5	28.5	28.5
Required EIRP (dBW)	68.4	75.9	63.2	62.3
Theoretical Radiant Elements (10)	3008	8318	1928	1738
SystemVue Radiant Elements (12)	3969	9689	2128	1976

**Table 5:** Area used by parabolic antennas in transmission.

LEO Constellation	Diameter (m)	Area (m^2^)	Above Ground Level (m)
SpaceX	1.5	1.8	1.95
Telesat	1.5	1.8	3.2
OneWeb	3.5	9.6	4.55
Amazon	2.4	4.5	-

**Table 6:** Area used by phased array antennas in transmission.

LEO Constellation	Diameter (m)	Area (m^2^)
SpaceX	1.06	0.88
Telesat	1.56	1.92
OneWeb	0.78	0.47
Amazon	0.75	0.44

**Table 7:** Sensitivity calculations for each constellation using Equation (17).

Satellite	Path Distance (km)	FSPL (dB) (18)	Atm Losses (dB)	Sensitivity (dBm) (17)
SpaceX	2439	−185.8	2	−113
Telesat	1684	−182.5	2	−102
OneWeb	1504	−181.6	2	−114
Amazon	590	−173.4	2	−106

**Table 8:** Reception antenna size estimation for hexagonal configuration.

Constellation	SpaceX	Telesat	OneWeb	Amazon
Operation Frequency (GHz)	18.55	19	18.55	19
Required Gain (dB)	46.9	46.5	51.5	50.2
Theoretical Radiant Elements (16)	11,749	8512	33,885	25,119
SystemVue Radiant Elements (20)	8910	7956	26,791	19,927

**Table 9:** Area used by parabolic antennas in reception.

LEO Constellation	Diameter (m)	Area (m^2^)	Above Ground Level (m)
SpaceX	1.5	1.8	1.95
Telesat	1.5	1.8	3.2
OneWeb	3.5	9.6	4.55
Amazon	2.4	4.5	-

**Table 10:** Area used by phased array antennas in reception.

LEO Constellation	Aphotem (m)	Side (m)	Area (m^2^)
SpaceX	0.95	0.85	2.44
Telesat	0.91	0.82	2.22
OneWeb	1.6	1.5	7.6
Amazon	1.4	1.3	5.6

**Table 11:** Results’ summary.

	Transmission		Reception		
	EIRP (dBW)	Number of Elements	Sensitivity (dBm)	Gain (dB)	Number of Elements
SpaceX	68.4	4000	−113.1	46.9	8910
Telesat	75.9	9600	−102.5	46.5	7956
OneWeb	63.2	2150	−114.1	51.5	26,791
Amazon	62.3	1950	−106.9	50.2	19,927

## Abbreviations

The following abbreviations are used in this manuscript:

LEO	Low Earth Orbit
ADS	Advanced Design Software
EIRP	Effective Isotropic Radiated Power
SOTM	SATCOM On The Move
GEO	Geostationary Earth Orbit
IoT	Internet of Things
TT&C	Tracking, Telemetry and Commands
MEO	Medium Earth Orbit
MSS	Mobile Satellite Services
MIMO	Multiple Input Multiple Output
HTS	High Throughput Satellite
FCC	Federal Communications Commision
EM	Electromagnetic
UAN	User-Defined Antenna
RF	Radiofrequency
NF	Noise Figure
PCB	Printed Circuit Board
